# Nanofibrous Gelatin-Based Biomaterial with Improved Biomimicry Using D-Periodic Self-Assembled Atelocollagen

**DOI:** 10.3390/biomimetics6010020

**Published:** 2021-03-18

**Authors:** Sara Borrego-González, Matthew J. Dalby, Aránzazu Díaz-Cuenca

**Affiliations:** 1 Materials Science Institute of Seville (ICMS), Joint CSIC-University of Seville Center, C/Américo Vespucio 49, Isla de la Cartuja, 41092 Seville, Spain; sarbogo@gmail.com; 2 Center for Cell Engineering, Institute of Molecular, Cell and Systems Biology, CMVLS, University of Glasgow, Joseph Black Building, Glasgow G12 8QQ, UK; matthew.dalby@glasgow.ac.uk; 3 Networking Research Center on Bioengineering, Biomaterials and Nanomedicine (CIBER-BBN), 28029 Madrid, Spain

**Keywords:** atelocollagen, D-periodic collagen, nanofibrous gelatin, collagen-like biomaterials, TIPS, 3D cell culture

## Abstract

Design of bioinspired materials that mimic the extracellular matrix (ECM) at the nanoscale is a challenge in tissue engineering. While nanofibrillar gelatin materials mimic chemical composition and nano-architecture of natural ECM collagen components, it lacks the characteristic D-staggered array (D-periodicity) of 67 nm, which is an important cue in terms of cell recognition and adhesion properties. In this study, a nanofibrous gelatin matrix with improved biomimicry is achieved using a formulation including a minimal content of D-periodic self-assembled atelocollagen. We suggest a processing route approach consisting of the thermally induced phase separation of the gelatin based biopolymeric mixture precursor followed by chemical-free material cross-linking. The matrix nanostructure is characterized using field emission gun scanning electron microscopy (FEG-SEM), transmission electron microscopy (TEM), wide angle X-ray diffraction (XRD) and Fourier-transform infrared spectroscopy (FT-IR). The cell culture assays indicate that incorporation of 2.6 wt.% content of D-periodic atelocollagen to the gelatin material, produces a significant increase of MC3T3-E1 mouse preosteoblast cells attachment and human mesenchymal stem cells (hMSCs) proliferation, in comparison with related bare gelatin matrices. The presented results demonstrate the achievement of an efficient route to produce a cost-effective, compositionally defined and low immunogenic “collagen-like” instructive biomaterial, based on gelatin.

## 1. Introduction

The design of materials that mimic the extracellular matrix (ECM) is a challenge in tissue engineering. Efforts are currently devoted to improving key biomaterials’ features such as surface chemistry, topography and stiffness [1,2,3]. The ECM is a complex structure where cells reside, remodel and interact over a range of length scales to maintain tissue homeostasis, growth and repair. The structure at the nanoscale of the ECM provides a natural net of intricate nanofibers, collagen being one of the main protein components. Type I collagen is the most abundant collagen of the human body, for example, it forms more than 90% of the organic mass of bone [4]. The collagen basic molecule (tropocollagen) is characterized by its triple-helical structure of 300 nm in length formed by three polypeptide chains, which self-assemble into large fibrillar supramolecules [5]. Current models suggest that five of these collagen molecules assemble to form one initial fibril as the basic building block of larger fibrils with diameters of about 100 nm [6,7]. This arrangement produces an axial periodicity of 67 nm, so-called D-periodic spacing, which is characteristic of collagen type I materials [8,9] and can be clearly observed by electron microscopy [10]. The resulting organization and amino acid placement produce a spatial imprint of specific epitopes (ligands) and chemical functional groups within the fibrillar ECM that cells may respond to.

Mammalian cells attach to ECM motifs via integrins, heterodimeric transmembrane proteins consisting of α and β subunits. On the extracellular side, integrins recognize specific amino acid sequences, allowing them to adhere to various components of the ECM. In addition to RGD, the most prevalent peptide motif, other collagen type I adhesive peptide motifs include GFOGER and DGEA [11,12]. Integrins α1β1 and α2β1 are the major integrin collagen receptors and recognize the same specific GFOGER amino acid sequence in triple helical collagens [13]. Hence, α2β1 integrin promotes the integrin-mediated formation of long cellular projections typically induced by fibrillar collagen and the α2β1 integrin-specific GFOGER peptide triggers the activation of focal adhesion kinase and alkaline phosphatase in osteoblastic cells, both implicated in the osteoblast differentiation pathway [14,15]. Specific periodicity similar to the collagen pattern of 63 nm (±5 nm), achieved in synthetic helical nano-ribbons, has induced much more specific cell adhesion and greater commitment to the osteoblast lineage than twisted nanoribbons with a periodicity of 100 nm helping to demonstrate that nanoscale biomimicry is important [16].

Although collagen-based biomaterials extracted from animal sources has been proven effective in a wide variety of medical applications [17,18], present batch-to-batch variability and other drawbacks such as high degradation rates, poor processability, high costs of manufacturing and the possibility of diseases transfection or immune responses [19]. Gelatin instead, that mimics collagen chemical composition including linear RGD cell adhesive motifs, has been recognized as a GRAS (generally regarded as safe) material by the FDA [20,21], and can be fabricated in the form of permeable fibrillar structures [22]. However, the gelatin is a denatured protein and derived materials lack the characteristic D-staggered array (D-periodicity), which is an important cue in terms of cell recognition and adhesion properties.

To overcome these limitations, atelocollagen, a form of highly purified collagen from which the telopeptides have been enzymatically removed, is a very promising candidate for application in tissue engineering and regeneration therapies [23,24]. Atelocollagen products in the monomeric form are commercially available and can reproducibly self-assemble in vitro into organized fibrils of D-periodicity [25,26]. The advantages in terms of biocompatibility and reduced immunogenicity, are encouraging very promising research using atelocollagen of different sources and processing forms. Hence, porcine atelocollagen gel beds have been shown to improve human mesenchymal stem cells (hMSCs) chondrogenic differentiation [27], and fibril coatings using Bester sturgeon atelocollagen and MC3T3-E1 cells are proven as interesting tools for in vitro studies [28]. Three dimensionally cultured adipose tissue-derived mesenchymal stem cells (ADMSCs), on honeycomb scaffolds of atelocollagen from cattle, have demonstrated good biocompatibility with host cardio tissue in in vivo [29], and atelocollagen suspended in mesenchymal stem cells (MSCs) secretome implants enhanced the migration of endogenous stem cells in the rat calvarial bone defect model [30]. Besides, injectable forms of atelocollagen are being assayed in the clinic and outcomes recently reported for meniscal root repair [31] and gingival soft tissue regeneration [32].

However, the use of monomeric atelocollagen for the fabrication of collagen-based biomaterials in the solid form is not widespread. Although, the casting and freeze-drying of atelocollagen formulations have been assayed [26,33], new synthesis and processing route strategies remain relatively unexplored. In this respect, the presented work brings forward the use of atelocollagen as a functional component to achieve gelatin-based biomaterials with enhanced biomimicry.

**Scheme 1:**
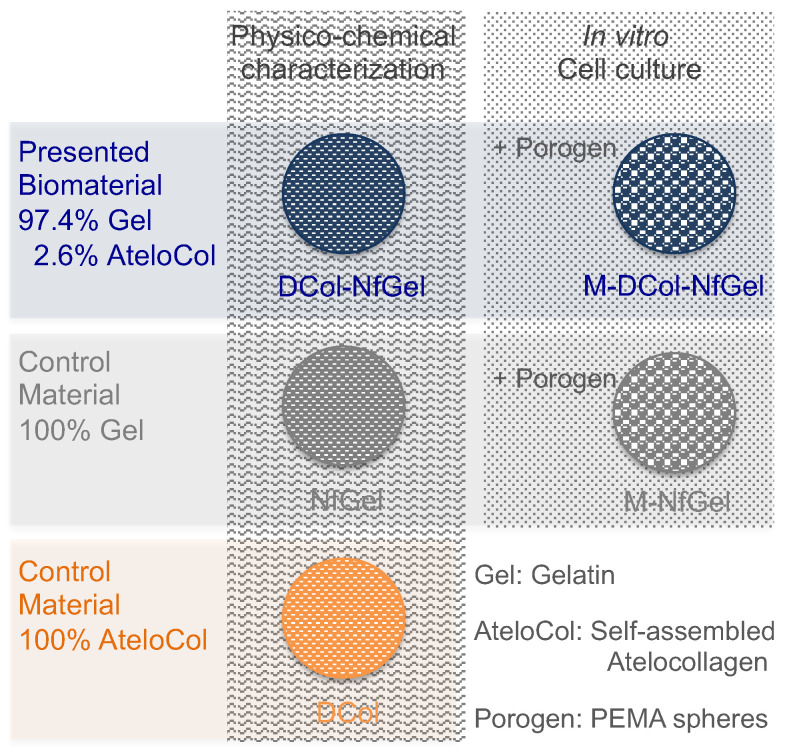
Material pieces processed for the physico-chemical and the in vitro cell culture studies, and nomenclature used. PEMA: Polyethylmethacrylate.

The purpose of the work is to achieve a simple yet scalable process-controlled nano-fibrous “collagen-like” material based on gelatin, and to demonstrate its improved functionality in terms of cell response. The presented cost-effective processing route consists of incorporating a minority amount of D-periodic self-assembled atelocollagen, as functional ligand, within the nanofibrous gelatin using a combination of thermally induced phase separation (TIPS) and porogen-leaching. Our hypothesis is that nanofibrous gelatin matrices with improved biomimicry can be reproducibly processed by minimal integration of specific triple helical collagen motifs using D-periodic atelocollagen fibrils. To prove this, a series of materials in the form of solid pieces, including the presented matrix material, the D-periodic structured atelocollagen functionalized nanofibrous gelatin (DCol-NfGel), and two single-component (bare) control materials of D-periodic structured atelocollagen (DCol) and gelatin (NfGel), respectively, were processed to carry out appropriate characterization and cell response evaluation. Scheme 1 displays the types of material prepared pieces and nomenclature used throughout the work.

## 2. Materials and Methods

### 2.1. D-Periodic Collagen Fibrils Precursor Synthesis

A collagen type I ((atelo)collagen) solution (354231, BD Biosciences, Corning, Bedford, MA, USA) in 0.01 M hydrochloric acid, HCl, at a concentration of 3.1 mg mL^−1^ was used. Fibril formation was achieved using 125 µL of 150 mM phosphate buffer (Na_2_HPO_4_/KH_2_PO_4_) and 500 µL of the atelocollagen acid solution. The pH of the mixture solution was adjusted to 7.4 and incubated at 34 °C for 24 h. After incubation time, a washing protocol was performed to eliminate residual salt impurities and a Bradford assay was used for quantifying final collagen content of the as-prepared fibril suspension. Full details of the procedure can be found in a recent publication by the authors [26].

### 2.2. D-Periodic Collagen Solid Pieces (DCol) Processing

Single component (bare) D-periodic collagen matrices (DCol) were prepared as control samples. The fibril suspension precursor was shaken and 100 μL portions were cast into the wells of a 96-well polyethylene plate (655161, Greiner Bio-one, Frickenhausen, Germany) and frozen at −80 °C. Then, the plate was placed into a freeze-drier for lyophilization (Epsilon 2-4 LSC CHRIST, Osterode, Germany) at −30 °C and 0.04 mbar for 10 h, followed by a gradual temperature increasing to 20 °C. Finally, a dehydrothermal treatment (DHT) of 150 °C and 10 mbar for 20 h was applied using a vacuum oven (VO 200 Memmert, Schwabach, Germany) for the crosslinking [22].

### 2.3. D-Periodic Collagen—Nanofibrous Gelatin (DCol-NfGel) Pieces Processing

**Figure 1:**
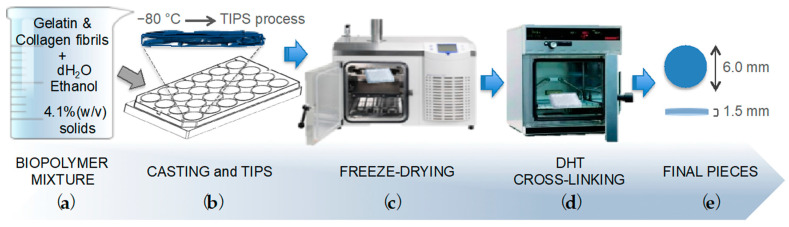
Nanofibrous biomaterial processing route: (**a**) Biopolymer mixture precursor composition; (**b**) pieces shaping and thermally induced phase separation (TIPS); (**c**) lyophilization process; (**d**) dehydrothermal treatment (DHT) cross-linking; (**e**) final pieces dimensions.

The preparation of the nanofibrous matrices using the thermally induced phase separation (TIPS) technique was adapted from the method reported by Liu et al. [34], as well as a previous work carried out by the authors, using the single-component gelatin system, on the influence the experimental processing conditions in the final material microstructure [22]. First, the D-periodic collagen precursor, obtained as described in Section 2.1, was preconditioned (agitated) for 1 h using 1300 rpm magnetic stirring at 34 °C. In parallel, 0.04 g of gelatin were mixed with 200 μL dH_2_O and stirred using 500 rpm at 45 °C for 45 min. Then, 200 μL ethanol (UN1170, AnalaR Normapur) were added to the gelatin mixture and stirred for other 30 min. After this time, the temperature was lowered to 34 °C, and 300 μL of D-periodic collagen suspension, 200 μL of ethanol and 100 μL of dH_2_O were added to the mixture, which was kept in magnetic stirring for 45 min at 500 rpm (Figure 1a). A water/ethanol (*v*/*v*) final ratio of 60/40 is used considering the 300 μL of collagen fibril suspension as water volume. Then, 100 μL of the gelatin-collagen mixture (total solids content 4.1% (*w*/*v*) consisting of 97.4 wt.% gelatin and 2.6 wt.% collagen) were cast into the wells of a polystyrene 96-well plate (655161, Greiner Bio-one), which was previously placed on a prewarmed thermoblock at 36 °C (AccuBlockTM Digital Dry Baths, Labnet, Woodbridge, NJ, USA) to control the casting temperature (Figure 1b). Then, the 96-well plate containing the mixture was immediately frozen at −80 °C for 5.5 h allowing the TIPS process. Furthermore, solvent exchange using ethanol and acetone was carried out at −20 °C. Then, the pieces were placed into a freeze-drier (Epsilon 2-4 CHRIST) at −30 °C and 0.04 mbar for 10 h, followed by a gradual temperature increase up to 20 °C (Figure 1c). Finally, obtained pieces were introduced inside a vacuum oven (VO 200 Memmert, Schwabach, Germany) to carry out a DHT cross-linking process (Figure 1d). The oven parameters were set to 10 mbar at 150 °C for 20 h. Final solid pieces of 6 mm diameter and 1.5 mm thickness (Figure 1e) were stored in a desiccator until use.

Single component (bare) nanofibrous gelatin matrix (NfGel) was also prepared as control material using 5.4% (*w*/*v*) gelatin in water/ethanol (*v*/*v*) of 50/50 [22]. This precursor mixture was first conditioned at 40 °C and 500 rpm for 1 h, and then the temperature was raised to 45 °C and was maintained for 1 h at 1100 rpm. Finally, 100 μL of the mixture were cast into a 96-well plate which was placed on a pre-warmed thermoblock at 38 °C. The casted mixture was then placed at −80 °C, ethanol and acetone solvent exchange treated, freeze-dried and DHT cross-linked using similar conditions as detailed above.

### 2.4. Macroporous—DCol-NfGel (M-DCol-NfGel) Pieces Processing

Pieces with additional macropore cavities were prepared to perform the cell culture experiments. Atelocollagen monomers self-assembly, TIPS and porogen-leaching were combined to obtain macroporous DCol-NfGel (M-DCol-NfGel) matrix pieces. Furthermore, 0.035 g of polyethylmethacrylate (PEMA) spheres (2043, Elvacite) of 140–220 μm particle size, kindly provided by Lucite International, were deposited into each of the wells of a 96-well polypropylene plate (655201, Greiner Bio-one, Frickenhausen, Germany). The plate was vigorously tapped to pack the spheres and placed on a prewarmed thermoblock at 36 °C. Furthermore, 100 μL of the collagen/gelatin mixture precursor (Section 2.3) were cast onto the porogen spheres. Then, the plates were frozen at −80 °C overnight allowing the TIPS process. Furthermore, cold ethanol at −20 °C was added to the samples and stored in the freezer at −20 °C for 20 h. After that, the materials were freeze-dried and cross-linked following the same protocol as described in the previous sections. Then, the PEMA spheres were removed from the matrices by dissolution in ethanol at room temperature. The pieces were soaked in ethanol at the proportion of 1 piece/mL for 24 h, threefold. Finally, the matrices were freeze-dried for a second time following the same protocol as before. Final pieces were 5.2 mm diameter and 1.8 mm height. Similarly, macroporous pieces of bare gelatin, macroporous-nanofibrous gelatin (M-NfGel) were also prepared as control material.

### 2.5. Scanning Transmission Electron Microscopy (STEM) and Conventional Field Emission Gun Scanning Electron Microscopy (FEG-SEM) Observations

STEM observations were performed after negative staining, using phosphotungstic acid, and carbon coating of the samples as detailed previously [26,35]. Samples were examined using a HITACHI S-4800 field emission gun scanning electron microscope (FEG-SEM) working in transmission mode and operating at an accelerating voltage of 30 kV. The FEG-SEM observations of the matrices were carried out using a working voltage of 2 kV. Cross-sections of the pieces were previously coated with carbon using an EMITECH K950 carbon evaporator and 60 A of voltage.

### 2.6. Transmission Electron Microscopy (TEM) Observations

Samples were first fixed using 1.6% glutaraldehyde in cacodylate buffer (0.1 M and pH 7.4) for 1 h at room temperature. Post-fixation staining was done using 1% OsO_4_ with cacodylate buffer (0.1 M and pH 7.4) and 2% uranyl acetate, dehydrated in acetone and infiltrated in Spurr resin (18306-4221, Ted Pella). Then, ultrathin sections (70 nm) were cut using automated electronic microscopy tissue processing Leica EM TP (Leica Microsystems, Wetzlar, Germany) and were deposited on grids. Samples were examined using a Philips CM-200 operating at an accelerating voltage of 80 kV.

### 2.7. Physico-Chemical Characterization

The total porosity (*P*) of the pieces was calculated by *P* = 1 − (ρ_matrix_/ρ_solid_), where ρ_solid_ was estimated using the proportions of each component and solid density values for gelatin (ρ_gelatin_ = 1.037 g cm^−3^) [36] and collagen (ρ_collagen_ = 1.343 g cm^−3^) [37]. X-ray diffraction (XRD) analysis was performed with a PANalytical X’Pert PRO diffractometer using Cu-Ka radiation (0.154187 nm) and step size of 0.02 and 800 s exposure time. The Fourier-transform infrared spectroscopy (FT-IR) analyses were carried out using a JASCO FT/IR 6200 IRT 5000 spectrometer in transmission configuration. The material pieces were cut finely and dispersed into pellets of potassium bromide (KBr) and compressed under 8 tonnes to obtain disks. The spectra were recorded in the absorption mode at 4 cm^−1^ intervals in the 4000–300 cm^−1^ range. The water uptake ability of the macroporous matrix materials, W_uptake_, was calculated by W_uptake_ = (W_w_ − W_d_)/W_d_, where W_d_ is the mass of the dried piece and W_w_ is the mass after the different immersion times using 4 mL of phosphate buffered saline (PBS), pH 7.4, per piece at 36.5 °C.

As native D-periodic reference control material, a purified collagen sponge (PCS) (extracted and purified native bovine skin collagen, kindly provided as a gift by LABRET-UMA Laboratory and referred to as Nimni, M.E.; US patent 5374539) [38], was prepared appropriately and used for the characterization analysis of Section 2.5 and Section 2.7.

### 2.8. In Vitro Cell Culture Study

MC3T3-E1 mouse pre-osteoblast cells from the European Collection of Authenticated Cell Cultures (ECACC), catalogue number 99072810, were cultured in minimum essential medium, alpha modification (alpha-MEM; Sigma–Aldrich, Steinheim, Germany) supplemented with 10% fetal bovine serum (FBS), 100 U/mL penicillin, 100 mg/mL streptomycin and 2 mM L-glutamine at 37 °C in a humidified atmosphere with 5% CO_2_.

Human mesenchymal stem cells (hMSCs) from adipose tissue (hMSC-AT, PromoCell) were maintained in Dulbecco’s modified Eagle Medium (DMEM) (41965039, Gibco, Thermo Fisher Scientific, Waltham, MA, USA) supplemented with 4.5 g L^−1^ glucose, 100 μM sodium pyruvate (S8636 Sigma-Aldrich), 1 mM L-glutamine, 10% fetal bovine serum (FBS) (10500, Gibco) and 100 U mL^−1^ penicillin/streptomycin (P0781, Sigma-Aldrich). Cultures were kept at 37 °C and 5% CO_2_ in a humidified atmosphere.

Mouse embryogenic stem cells (mESCs) ES-D3 cell line (American Type Culture Collection, ATTC) were subcultured in embryogenic stem cells qualified DMEM medium (SLM-220-M, Millipore) supplemented with 10% FBS ES-qualified (ES-009-B, Millipore), 1% nucleosides (ES-008-D, Millipore), 1% penicillin/streptomycin (TMS-AB2-C, Millipore), 1% non-essential amino acids (100×) (TMS-001-C, Millipore), 1% L-glutamine solution (100×), 200 mM (TMS-002-C, Millipore), 1% 2-mercaptoethanol (ES-007-E, Millipore), 5 mL LIF (ESGRO 105 U/mL) (ESG1107, Millipore). Cultures were kept at 37 °C and 5% CO_2_ in a humidified atmosphere.

Cell experiments were performed on matrix pieces placed in 96-well plates. The material pieces were previously sterilized in 100% ethanol for 48 h at 37 °C. Decreasing ethanol in PBS solutions was used to rehydrate the materials, which were finally incubated overnight in PBS. After that, cell media was added into the wells and 1 × 10^4^ cells were seeded on each material piece.

Quant-iT PicoGreen dsDNA Assay Kit (P7589, Invitrogen) was used to quantify the cell number attached to the matrices. Furthermore, 100 μL lysis buffer (10 Mm Tris, 1mM EDTA and 0.2% Triton ×100) was added for 10 min at room temperature and shaken at 100 rpm. After incubation, 0.1 mL of lysate was transferred to a clean 96-well plate and kept at −80 °C until use. Picogreen kit assay was used according to the manufacturer’s protocol to quantify double-stranded DNA within the samples. The plate was incubated in the dark for 5 min, and then fluorescence emission at λ = 538 nm was measured under excitation at λ = 480 nm on an Infinite M200 PRO (TECAN) plate reader. Live cells were stained with calcein acetoxymethyl ester (Calcein AM, C3099, Molecular ProbesTM) (1:5000) and incubated for 15 min at 37 °C. Fluorescence images were obtained using a Nikon Eclipse 80i microscope. Phalloidin staining was performed on hMSCs washed thrice after using a fixative solution of 4% formaldehyde in PBS for 15 min. Rhodamine phalloidin (R415, Life Technologies) (1:100) was added for 1 h to stain actin and it was washed thrice. Finally, mounting medium for fluorescence, Vectashield^®^ with DAPI (H-1200, Vector Laboratories, Burlingame, CA, USA) was used and the images were obtained using the Nikon Eclipse 80i microscope.

### 2.9. Statistical Analysis

Data are expressed as the mean ± standard deviation (SD). All experimental groups had a sample size of at least *n* = 3. Statistical analysis was performed using the two population Student’s t-test assuming unequal variances. Differences were considered significant when *p* < 0.05 (*), *p* < 0.01 (**) and *p* < 0.001 (***).

## 3. Results

### 3.1. Synthesised D-Periodic Collagen Fibrils STEM Characterization

**Figure 2:**
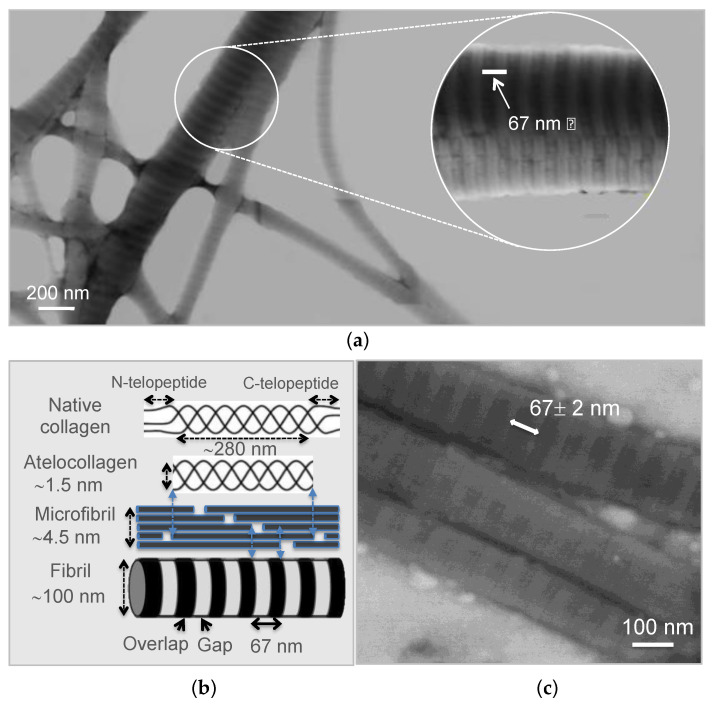
Collagen fibrils scanning transmission electron microscopy (STEM) characterization: (**a**) Negatively stained sample micrograph of the native reference material, the purified collagen sponge (PCS), showing the typical D-periodic contrast pattern of 67 nm; (**b**) schematic draw of hierarchical atelocollagen molecules self-assembly; (**c**) negatively stained sample micrograph of the synthesized collagen fibrils precursor.

The STEM characterization performed of type I native collagen reference material, the purified collagen sponge (PCS), is shown in Figure 2a. In comparison, this work self-assembled collagen fibrils obtained from atelocollagen (Figure 2c), displaying the characteristic D-periodic banding contrast pattern of 67 ± 2 nm [5,9]. Synthesized fibril suspension precursor of 3.57 ± 0.05 mg mL^−1^, as quantified by the Bradford assay, is formed of nanostructured fibrils of 150 ± 20 nm diameter average.

### 3.2. DCol-NfGel Matrix Processed Pieces Characterization

**Figure 3:**
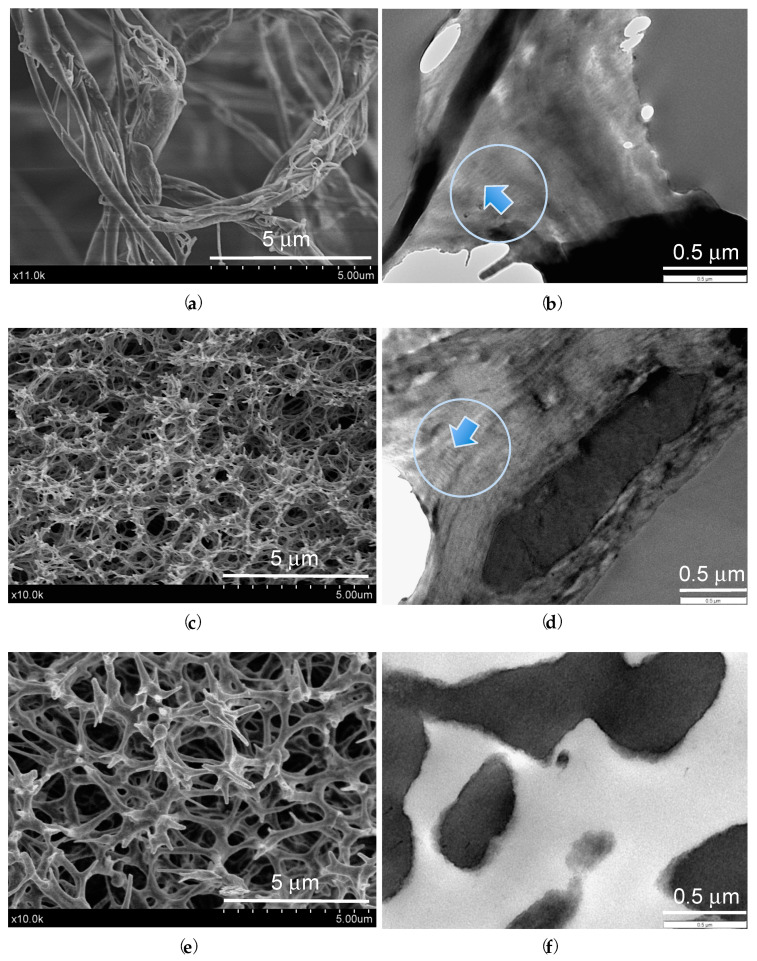
Electron microscopy characterization of the DCol-NfGel matrix and control materials, DCol and NfGel: (**a**) Cross-sectional area field emission gun scanning electron microscopy (FEG-SEM) image of DCol; (**b**) transmission electron microscopy (TEM) micrograph of DCol showing D-periodic patter resolution (arrow); (**c**) cross-sectional area FEG-SEM image of DCol-NfGel; (**d**) TEM micrograph of DCol-NfGel showing D-periodic patter resolution (arrow); (**e**) cross-sectional area FEG-SEM image of NfGel; (**f**) TEM micrograph of NfGel.

Pieces of 6.0 mm diameter and 1.5 mm thickness were obtained with total porosity of 92%. Cross-sectional area FEG-SEM images of the presented biomaterial, DCol-NfGel, and the control materials, the bare gelatin, NfGel, and the bare collagen, DCol, are comparatively displayed in Figure 3a,c,e. Micrograph of DCol-NfGel (Figure 3c) shows the formation of a homogeneous nanofibrillar structure featuring fibers of 77 ± 30 nm diameter and interconnected cavities of 0.58 ± 0.25 μm. For its part, the NfGel matrix (Figure 3e) of 90% porosity, shows a similar structure but thicker nanofibers of 170 ± 50 nm diameter and bigger cavities of 1.16 ± 0.36 μm. In this respect, it is important to note the difference of the total biopolymer content (w/v) used for the two type of materials, 5.4% (w/v) for NfGel and 4.1% (w/v) for DCol-NfGel. This small difference in the solids content variable could well significantly affect final nanofibrillar microstructure [22].

In comparison, FEG-SEM observations of the 99% porosity DCol matrix (Figure 3a) show more irregular morphology with frayed fibers and fibril diameters of 150 ± 92 nm, in good agreement with STEM analysis carried out on the collagen precursor suspension (Figure 2c). The characteristic D-periodic contrast pattern was confirmed for DCol-NfGel matrix using TEM (Figure 3d), similarly for the DCol material (Figure 3b). Interestingly, D-periodic pattern resolution of the TEM observations was high for the DCol-NfGel in comparison with the bare collagen, DCol, material (Figure 3b,d).

**Figure 4:**
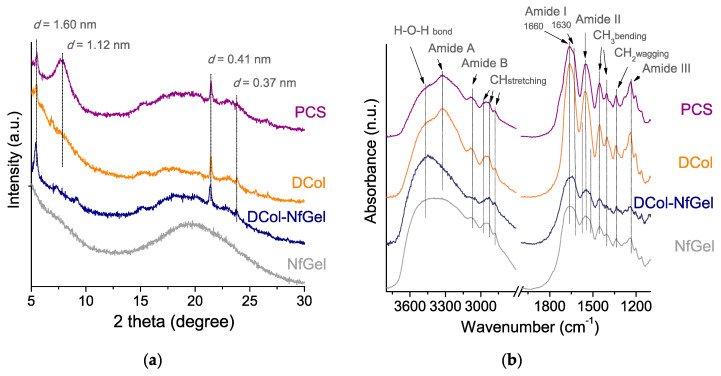
(**a**) X-ray diffraction patterns and (**b**) Fourier-transform infrared spectroscopy analyses of DCol-NfGel matrix and control materials.

The X-ray diffraction (Figure 4a) and FT-IR (Figure 4b) analyses of DCol-NfGel matrix were also performed in comparison with the two synthesized control materials DCol and NfGel, as well as the native collagen, PCS. PCS shows a semi-crystalline diffractogram where three 2 theta ranges can be differentiated: a first low peak at 2θ = 5.5° (*d* = 1.60 nm), a second wider peak at 2θ = 7.9° (*d* = 1.12 nm) and finally, a broad band in the range of 2θ = 12–27°. This broad band overlaps noticeable peaks at 2θ = 15.2° (*d* = 0.58 nm), 21.5° (*d* = 0.41 nm), 23.0° (*d* = 0.39 nm) and 23.9° (*d* = 0.37 nm). Observed peaks did not match with any International Centre for Diffraction Data (ICDD) card of inorganic compounds confirming actual correspondence with the collagen fibrils organized structure. Both, DCol and DCol-NfGel, showed crystalline peaks at 2θ = 5.5° and 2θ = 15.2°, 21.5°, 23.0° and 23.9°, similar to those detected in the native PCS material. Although, the broad peak at 2θ = 7.9° is not clearly shown in these synthesized matrices, an incipient intensity signal cannot be disregarded. The *d*-spacings of 1.60 nm and 1.12 nm, have been correlated to intermolecular lateral packaging of collagen fibrils [7,39]. Therefore, the peak at 2θ = 7.9° could well correspond to the higher supramolecular level. The other *d*-spacings of 0.41 nm and 0.37 nm match with the left-hand helix collagen chain *d*-spaces [39,40,41,42]. As expected, the bare gelatin NfGel matrix did not show any diffraction peak besides the typical amorphous halo from 2θ = 12° to 2θ = 29°. These results are indicative that the D-periodic structure is a constitutive structural feature of the atelocollagen-processed matrices. Especially relevant, is the high crystallinity obtained for DCol-NfGel considering its low collagen content of 2.6 wt.%.

Normalized FT-IR spectra analyses presented in Figure 4b, exhibit amide bands of N–H stretching at ~3326 cm^−1^ (Amide A), C–H stretching at ~3098 cm^−1^ (Amide B), C=O stretching at 1635 cm^−1^ (Amide I), N–H deformation at ~1540 cm^−1^ (Amide II), N–H deformation at ~1237 cm^−1^ (Amide III) and also the C–H, CH_2_– and CH_3_– stretching vibrations in the 2980–2830 cm^−1^ range [43,44,45,46]. The relative intensity absorbance bands of Amide I, II and III are higher for the collagen rich materials DCol and PCS, in comparison with NfGel and DCol-NfGel. The effect is associated with the collagen denaturation process occurring in gelatin biopolymers [43,45]. Likewise, correlated to collagen denaturation, the Amide A band at 3320 cm^−1^ ascribed to the N-H stretching vibration is clearly less resolved for NfGel and DCol-NfGel than for DCol and PCS [45].

### 3.3. M-DCol-NfGel Matrix Processed Pieces Characterisation

Pieces of 5.2 mm diameter and 1.8 mm thickness were obtained with total porosity values of 96% and 94%, respectively, for the M-DCol-NfGel and the control material M-NfGel. As expected, total porosity values for M-DCol-NfGel and M-NfGel matrices are higher than corresponding porogen-free processed counterparts.

**Figure 5:**
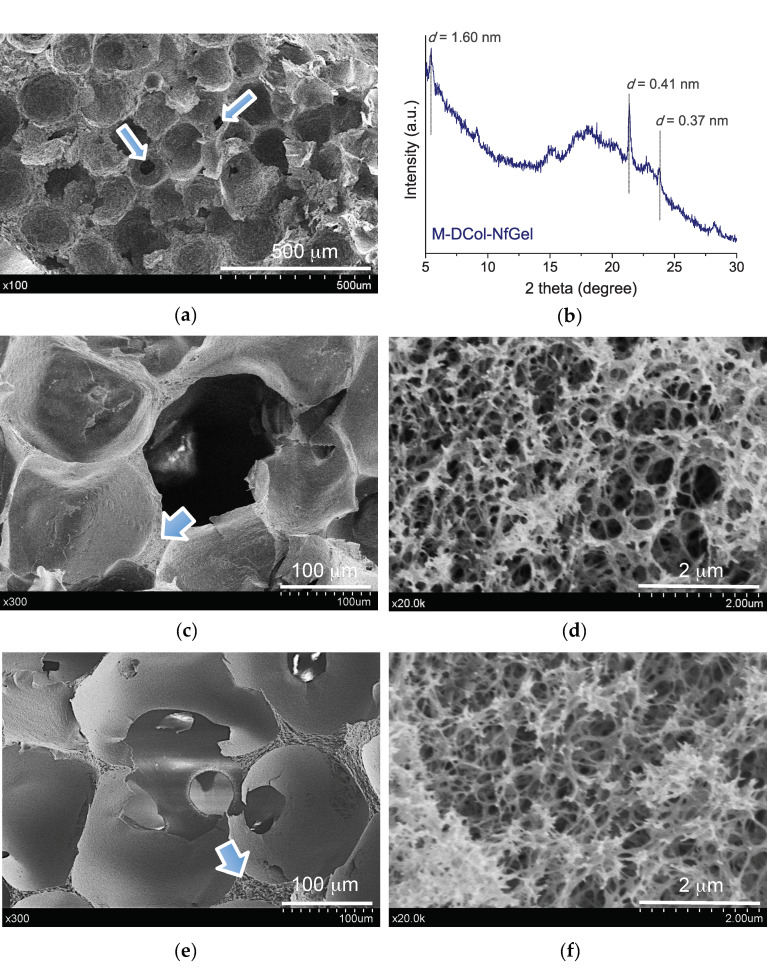
M-DCol-NfGel matrix processed pieces’ field emission gun scanning electron microscopy (FEG-SEM) and X-ray diffraction (XRD) characterization: (**a**) Low magnification FEG-SEM cross-sectional area image of M-DCol-NfGel showing macropore interconnectivity (arrows); (**b**) M-DCol-NfGel XRD pattern; (**c**) M-DCol-NfGel cross-sectional area low magnification FEG-SEM image; (**d**) M-DCol-NfGel higher magnification FEG-SEM image of selected area pointed to by the arrow inset in micrograph (**c**); (**e**) M-NfGel control material cross-sectional area low magnification FEG-SEM image; (**f**) M-NfGel higher magnification FEG-SEM image of selected area pointed to by the arrow in micrograph (**e**).

Spherical cavities of 140 ± 30 μm size showing neighbor macropore interconnectivity and biopolymer nanofibers of 60 ± 20 nm sizes were measured in the macropore walls for both materials as they are distinguishable in FEG-SEM micrographs in Figure 5. The XRD analysis of M-DCol-NfGel (Figure 5b) showed sharp peaks at 2θ = 5.5°, 21.5° and 23.9°, indicating similar collagen fibril structure definition as for the related porogen-agent free processed, DCol-NfGel, matrix.

**Figure 6:**
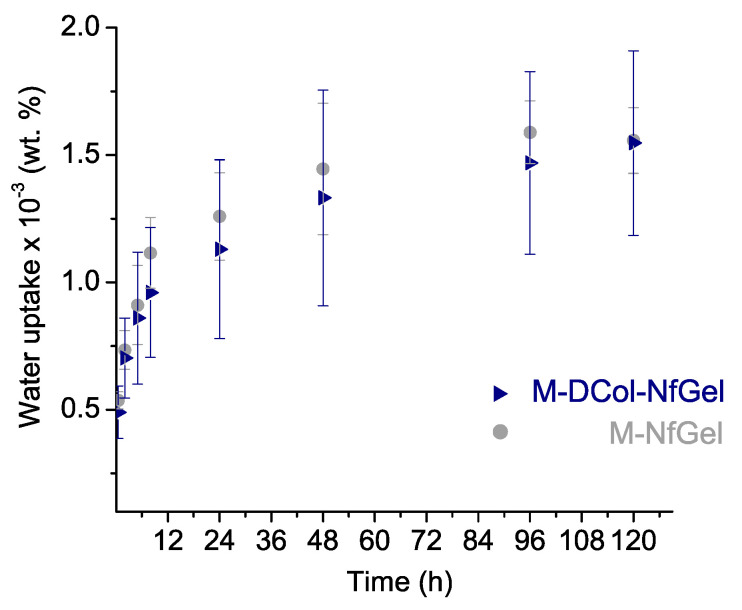
Water uptake ability as a function of time of M-DCol-NfGel matrix and control material, M-NfGel.

The water uptake ability for the macroporous matrix materials suggests good wetting and permeability performance for both materials (Figure 6). Values of 1560 and 1550 wt.% water uptake for M-NfGel and M-DCol-NfGel, respectively, were measured after 96 h water immersion (Figure 6). Although both materials show quite similar profiles with time, the value at 120 h for M-NfGel is slightly lower than the previous one at 96 h. This result might indicate not only the beginning of M-NfGel degradation, which is normal for a slightly crosslinked protein, but also that M-DCol-NfGel is more stable.

### 3.4. In Vitro Cell Culture Studies

#### 3.4.1. MC3T3 Cells Viability

**Figure 7:**
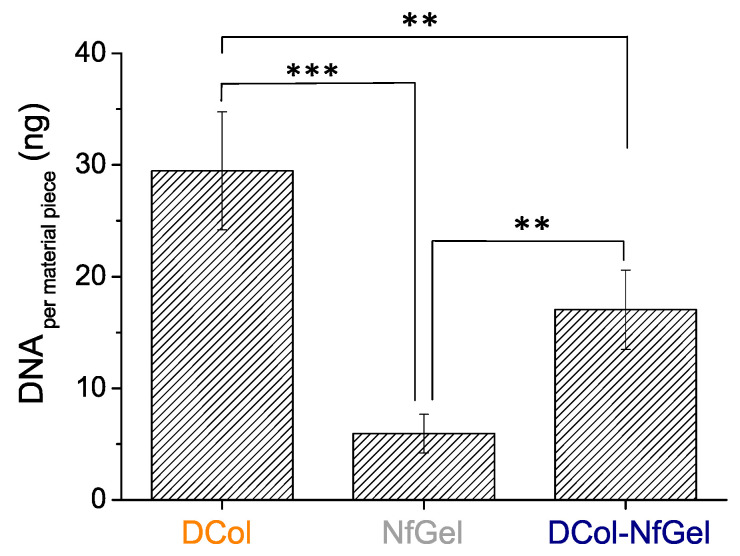
MC3T3-E1 cells’ DNA quantification after 48 h culture on DCol-NfGel matrix and control materials, DCol and NfGel. Statistically significant differences marked as ** *p* < 0.01, *** *p* < 0.001.

DNA quantification after 48 h was measured for DCol-NfGel in comparison with the two control materials, NfGel and DCol (Figure 7). As expected, DCol contains the highest number of cells, particularly in comparison with the bare gelatin matrix NfGel (*p* < 0.001). However, the results indicate that NfGel functionalization using only 2.6 wt.% of D-periodic atelocollagen produces a greater than 50% increase of quantified cells (*p* < 0.01).

#### 3.4.2. hMSCs Viability

**Figure 8:**
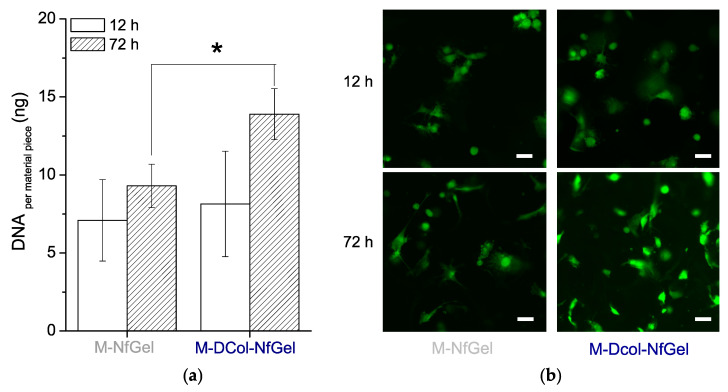
hMSCs culture on M-DCol-NfGel matrix and control material, M-NfGel: (**a**) DNA quantification, statistically significant differences marked as * *p* < 0.05; (**b**) calcein AM staining, the scale bars indicate 100 μm.

Processed macro-porous matrices, M-DCol-NfGel and control M-NfGel, were cultured using hMSC cells. DNA quantification after 12 and 72 h culture are plotted in Figure 8a. The plot show that values of 13.9 ± 1.6 ng and 9.3 ± 1.4 ng were measured, respectively, for M-DCol-NfGel and M-NfGel after 72 h, indicating that at this time culture DNA values for M-DCol-NfGel were significantly higher compared to NfGel (*p* < 0.05). These data suggest that hMSCs grow faster on the M-DCol-NfGel. Figure 8b shows calcein AM staining images of the hMSCs (live cells green stained cytoplasm) seeded on both matrices after the 12 and 72 h of culture. In good agreement with DNA quantification, not only higher number of cells but also more widespread morphology were observed after 72 h on M-DCol-NfGel in comparison with the M-NfGel matrix.

**Figure 9:**
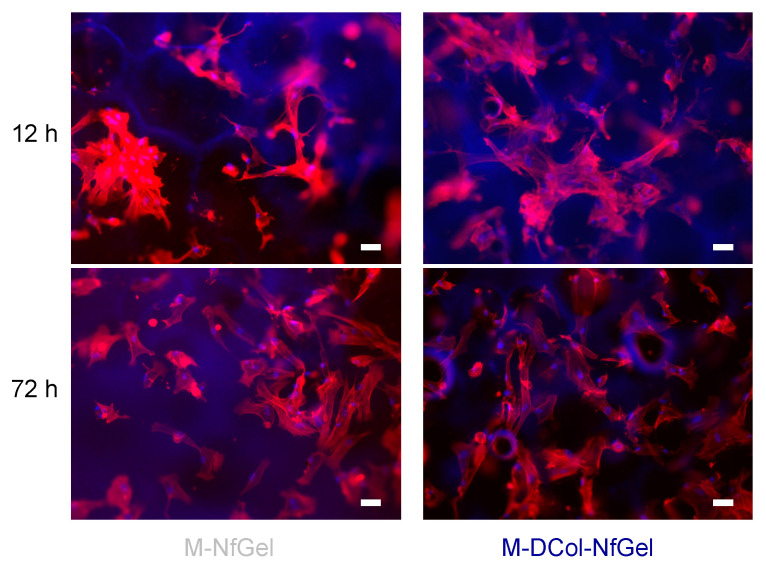
hMSCs culture on M-DCol-NfGel matrix and control material, M-NfGel: Fluorescent microscopy images showing nuclei in blue (DAPI) and actin in red (phalloidin), the scale bars indicate 100 μm.

Besides, the actin staining (Figure 9) demonstrates that cells were well spread on both matrices, particularly for the M-DCol-NfGel where a well-organized actin cytoskeleton was clearly observed within the first 12 h. The images show cells in different planes and some of them are clearly placed inside of the matrix cavities. This observation helps to indicate that the material porosity and interconnectivity is large enough for cells to migrate through.

#### 3.4.3. mESCs Viability

**Figure 10:**
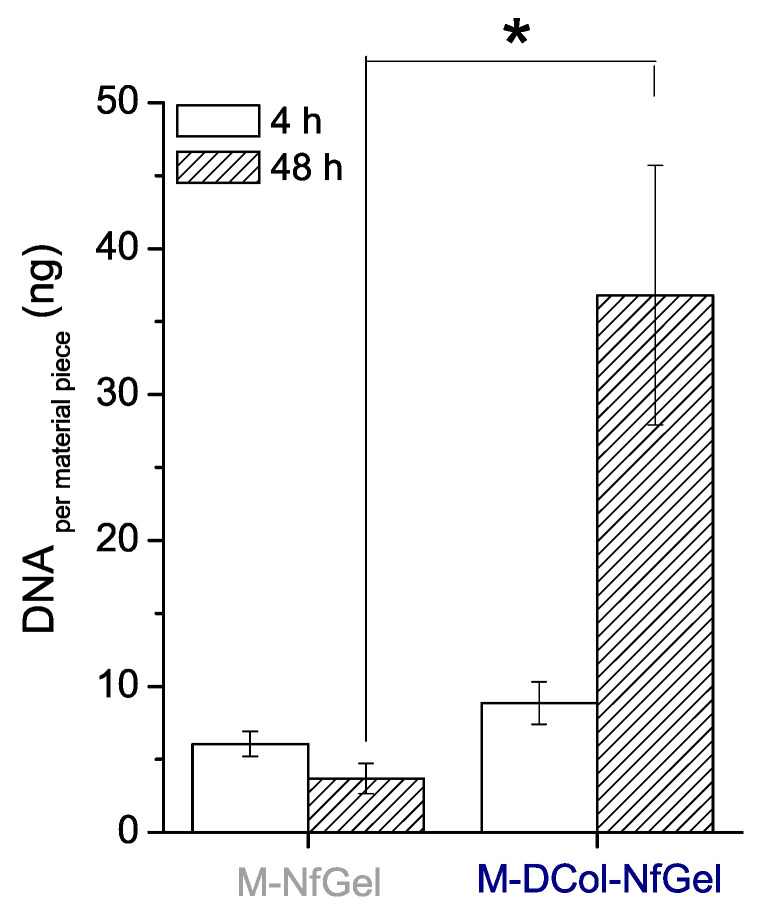
mESCs culture DNA quantification after 48 h on M-DCol-NfGel matrix and control material, M-NfGel. Statistically significant differences marked as * *p* < 0.05.

Figure 10 present the mESCs culture on the open macroporous M-DCol-NfGel matrix and bare gelatin matrix control material M-NfGel, after 4 and 48 h. DNA quantification results in Figure 10, indicate that after 48 h, M-DCol-NfGel matrices showed significant DNA increases to 36.8 ± 8.9 ng, whereas no growth was measured for cells on M-NfGel. A value of only 3.7 ± 1.1 ng, was measured for M-NfGel after 48 h culture.

## 4. Discussion

In this work, a nanofibrillar gelatin-based biomaterial has been fabricated and successfully functionalized using D-periodic collagen nanofibers synthesized from atelocollagen. The related macroporous material, M-DCol-NfGel, of ~140 μm interconnected cavities and pore walls of nanofibrillar structure, has also been processed and proved reproducible in the solid form, showing D-periodic features, being stable after hydration in wet media and biocompatible for 3D cell culture. M-DCol-NfGel matrix has been obtained using a processing route consisting of three-step procedure integration: monomeric atelocollagen self-assembly, TIPS process and porogen leaching. The TIPS technique is an easy, scalable and controllable procedure to obtain nanofibrous matrices [47,48], which is advantageous in comparison with other processing methods such as electrospinning [49,50,51], as it does not use toxic solvents. In this respect, electrospinning has been reported to cause collagen triple-helix degradation due to the use of fluoroalcohol-based agents [52,53,54,55,56]. Tsai et al. [57] reported that electrospinning of collagen results in the formation of denatured collagen showing no significant differences with electrospun gelatin matrices in terms of either cell attachment or proliferation of MG63 osteoblast-like cells. Besides, electrospinning usually produces densely packed nanofibers, which hinder cell infiltration and the accumulation of charge during the process limits the increase of thickness of the material piece [58].

Another interesting aspect of the presented material is the use of a toxin-free, DHT physical cross-linking method. Prevalent chemical cross-linking processes can induce cytotoxicity, inflammation, encapsulation, calcification and other unwanted effects [59,60]. Davidenko et al. [61] reported that carbodiimide cross-linking treatment may significantly decrease the content of carboxylic groups on glutamate and aspartate amino acid residues which can no longer interact with integrins leading to an important decrease of platelet attachments on highly crosslinked collagen biomaterials. In this sense, this work showed that DHT applied temperature and vacuum parameters preserve the material’s D-periodic structure, as demonstrated by TEM, XRD and FT-IR, whilst achieving required matrix stability and improved performance for cell culture.

Additionally, this work’s formulation using atelocollagen minimizes biomaterial antigenicity and represents an important advantage of M-DCol-NfGel. Atelocollagen is obtained after proteolytic elimination of telopeptides, which have been shown to trigger most of the collagen immunogenic reactions [62]. Hence, the proposed synthesis, based on highly purified atelocollagen monomers and gelatin, eliminates some of the important drawbacks in the production of collagen biomaterials based on animal extracted polymeric collagen such as the batch-to-batch variability and the risk of including impurities or infectious agents. Moreover, performed characterization of DCol-NfGel and its macropore counterpart, M-DCol-NfGel, confirms a robust D-periodic functionalization of the gelatin-based material using minimal atelocollagen content of only 2.6 wt.%.

**Figure A1:**
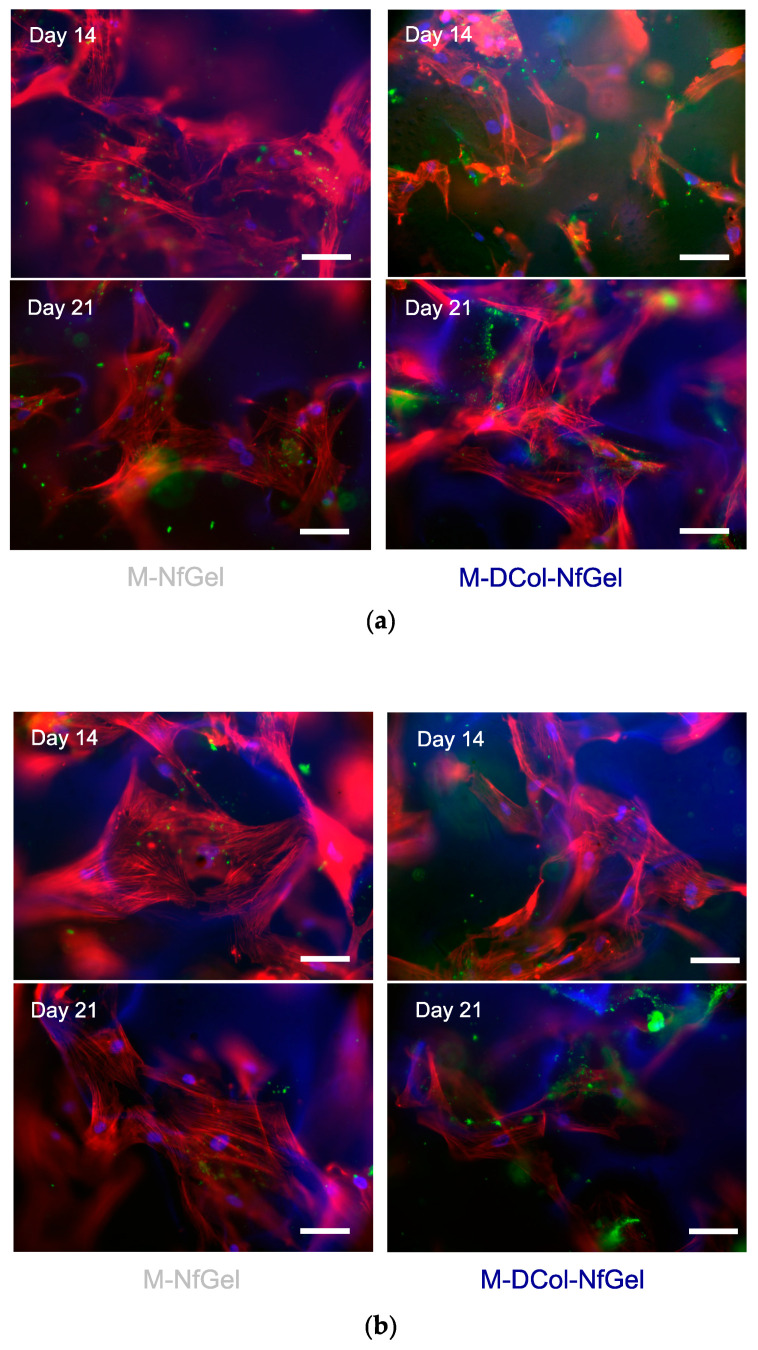
hMSCs culture on M-DCol-NfGel matrix and control material, M-NfGel, after 14 and 21 days: (**a**) Osteocalcin immunofluorescence; (**b**) Osteopontin immunofluorescence, the scale bars indicate 100 μm. The matrices’ ability to induce osteogenesis in the absence of additional induction media was studied using osteocalcin and osteopontin immunostaining after 14 and 21 days of cell culture. The figure displays stained cells (actin (red), nucleus (blue) and osteocalcin/osteopontin (green)). After 14 days, osteocalcin deposits were visible for both M-NfGel and M-DCol-NfGel matrices but with more abundance on M-DCol-NfGel. After 21 days of culture, green fluorescence for osteocalcin deposit increased on both, but, again, notably on M-DCol-NfGel samples. Osteopontin was only observed in deposits at day 21, but only M-DCol-NfGel showed significant deposits.

In fact, the MC3T3-E1 cells’ viability assays performed for functionalized DCol-NfGel, confirm a significant 50% increase of DNA with respect to the bare gelatin material. In turn, hMSCs and mESCs proliferation is also promoted in the M-DCol-NfGel functionalized matrix, in comparison to the gelatin single-component M-NfGel. Phalloidin and DAPI staining performed to hMSCs culture also indicate better spreading and well-organized actin cytoskeleton in M-DCol-NfGel matrix. Besides, complementary data of extended cell culture studies during 21 days (please see Appendix A) indicate that the matrices are stable without indication of major material degradation. Further, immunostaining for differentiation markers indicate production of discrete foci of osteocalcin and osteopontin deposition for D-periodic functionalized material after 14 and 21 days of culture, respectively, (Appendix A, Figure A1).

The viability promotion observed for the D-periodic functionalized matrices may well be correlated to the presence of collagen triple helical structures which provide the regular presentation of the integrin-binding amino acid sequence motif, GFOGER, along the fibril surface at a repetitive distance of ~67 nm [63]. GFOGER interacts with cells via the β1-containing integrins, α1β1, α2β1, α10β1 and α11β1 [61]. In this respect, hMSCs express α2β1 and type I collagen promotes proliferation and osteogenesis of hMSCs via activation of ERK and Akt through an integrin α2β1-independent pathway [64]. On the other hand, hMSCs are able to recognize gelatin RGDs through expression of α5β1 and αvβ3 [61,65]. Besides, α5β1 integrin has been shown to play an important role in hMSCs migration and osteogenic differentiation, while upregulating αVβ3 integrin can negatively regulate osteogenic differentiation [65]. In turn, mESCs express α2 and β1 integrin subunits and α5β1, which is the major functional integrin receptor, expressed on the cell surface of undifferentiated mouse ES-D3 cells [66,67]. Collagen I has been shown to regulate the self-renewal of mESCs through α2β1 integrin and discoidin domain receptor 1, (DDR1) [68] and to promote mESCs stemness and pluripotency when used in the feeder-free culture system of a collagen-grafted polyethersulfone nanofibrous scaffold [69]. Pimton et al. [67] indicated that the upregulation of α5β1 integrin and adhesion of ES-D3 cells to specific ECM molecules are linked to early stages of mESC commitment to meso-endodermal differentiation. Further, mESCs seeded in type I collagen scaffolds have shown not only to induce osteogenic differentiation of ESCs, but also to prevent ESCs from producing unwanted tumors when injected in vivo [70]. Other data indicating cell morphology and cell cycle alteration by collagen structure have been reported by Koohestani et al. [71]. These authors, demonstrated that using non-polymerized (monomeric) and polymerized (fibrillar) collagen, leiomyoma smooth muscle cells (LSMCs), had distinct morphologies on the different collagen matrices and their basal as well as platelet-derived growth factor (PDGF)-stimulated proliferation varied for these matrices.

Our results not only confirm D-periodic collagen structure relevance in ECM cell recognition and cell-matrix interaction but also indicate that nanofibrillar gelatin functionalization using a small content, 2.6 wt.%, of D-periodic collagen fibrils can significantly improve cell response performance. The presented data could well then be interesting to other types of polymeric matrix applications, particularly for those of synthetic nature, lacking natural cell recognition motifs.

This work presents a novel combination of techniques and synthesis strategies for the fabrication of an open-macroporous, nanofibrillar, collagen-like biomaterial, M-DCol-NfGel, which is suitable for cell culture in terms of stability for the wet culture media, cell infiltration properties and biocompatibility. Also of importance are its advantages concerning the precursor components safety and cost, the defined and reproducible formulation composition and the use of full toxin-free processing. Future work to characterize extended biodegradability and biocompatibility performance in vitro and in vivo, will provide valuable information to undertake further material processing optimization.

## 5. Conclusions

D-periodic collagen nanofibrils, synthetically self-assembled from atelocollagen monomers, were successfully incorporated into gelatin using TIPS processing to obtain functionalized nanofibrous matrices resembling natural tissue ECM structures. A porogen-leaching technique was also integrated to optimize higher macropore size as well as pore interconnectivity. XRD, FT-IR and TEM techniques confirmed the resolution of D-periodic collagen structure in final matrices after the processing, including DHT cross-linking treatment. Applied D-periodic collagen fibril functionalization promotes MC3T3 cells, hMSCs and mESCs viability and proliferation in vitro.

In summary, the presented fabrication strategy provides cost-effective, compositionally controlled and toxin-free collagen-like biomaterials based on gelatin, with enhanced biomimicry and bioactivity, through incorporation of minimal amounts of self-assembled atelocollagen.

## Data Availability

Not applicable.

## Appendix A

### hMSCs Differentiation on M-DCol-NfGel Matrix and Control Material, M-NfGel: Osteocalcin and Osteopontin Immunofluorescence

Cultivated hMSCs were fixed on the matrices using a solution of 4% formaldehyde (*v*/*v*) (F/1501/PB17, Fisher Chemical, Hampton, NH, USA) and 2% sucrose (*w*/*v*) (S/8600/53, Fisher Chemical) in PBS after 14 and 21 days of incubation time. After washing treatment with PBS, seeded matrices were incubated in permeabilizing buffer (sucrose, NaCl, MgCl_2_, HEPES (10756254, Fisher BioReagents) and triton (T8787, Sigma-Aldrich) in PBS). Then, the samples were incubated with 1% bovine serum albumin (BSA, 12841630, Fisher BioReagents) in PBS and then, with anti-osteocalcin (sc-73464, Santa Cruz Biotechnology, Inc., Dallas, TX, USA) and anti-osteopontin (sc-21742, Santa Cruz Biotechnology, Inc.) primary antibodies made in rabbit and diluted 1:50 in PBS/BSA containing phalloidin diluted 1:100. After 1 h incubation at 37 °C, the samples were washed thrice using 0.5% Tween 20 in PBS. Then, samples were incubated with biotinylated anti-rabbit secondary antibody made in horse and diluted 1:50 (BA-1100, Vector Laboratories) for 1 h at 37 °C in darkness. After washing treatment, fluorescein isothiocyanate (FITC)-conjugated streptavidin (SA-5001, Vector Laboratories diluted 1:50 in PBS/BSA was added for 30 min at 4 °C. Finally, after washing treatment, the samples were mounted with Vectashield^®^ with DAPI (H-1200, Vector Laboratories) and the images were obtained using the Nikon Eclipse 80i microscope (Nikon Instruments, Inc., Melville, NY, USA), Figure A1.
